# Comparative transcriptome and histological analyses provide insights into the skin pigmentation in Minxian black fur sheep (Ovis aries)

**DOI:** 10.7717/peerj.11122

**Published:** 2021-04-27

**Authors:** Xiaolei Shi, Jianping Wu, Xia Lang, Cailian Wang, Yan Bai, David Greg Riley, Lishan Liu, Xiaoming Ma

**Affiliations:** 1 College of Animal Science and Technology, Gansu Agricultural University, Lanzhou, Gansu Province, China; 2 Animal Husbandry, Pasture, and Green Agriculture Institute, Gansu Academy of Agricultural Sciences, Lanzhou, Gansu Province, China; 3 Key Laboratory for Sheep, Goat, and Cattle Germplasm and Straw Feed in Gansu Province, Lanzhou, Gansu Province, China; 4 Department of Animal Science, Texas A&M University, College Station, TX, USA

**Keywords:** Sheep, Skin, Pigmentation, RNA sequencing, Histology

## Abstract

**Background:**

Minxian black fur (MBF) sheep are found in the northwestern parts of China. These sheep have developed several special traits. Skin color is a phenotype subject to strong natural selection and diverse skin colors are likely a consequence of differences in gene regulation.

**Methods:**

Skin structure, color differences, and gene expression (determined by RNA sequencing) were evaluated the Minxian black fur and Small-tail Han sheep (*n* = 3 each group), which are both native Chinese sheep breeds.

**Results:**

Small-tail Han sheep have a thicker skin and dermis than the Minxian black fur sheep (*P* < 0.01); however, the quantity of melanin granules is greater (*P* < 0.01) in Minxian black fur sheep with a more extensive distribution in skin tissue and hair follicles. One hundred thirty-three differentially expressed genes were significantly associated with 37 ontological terms and two critical KEGG pathways for pigmentation (“tyrosine metabolism” and “melanogenesis” pathways). Important genes from those pathways with known involvement in pigmentation included *OCA2 melanosomal transmembrane protein* (*OCA2*), *dopachrome tautomerase* (*DCT*), *tyrosinase* (*TYR*) and *tyrosinase related protein* (*TYRP1*), *melanocortin 1 receptor* (*MC1R*), and *premelanosome protein* (*PMEL*). The results from our histological and transcriptome analyses will form a foundation for additional investigation into the genetic basis and regulation of pigmentation in these sheep breeds.

## Introduction

Sheep are important fiber and fur producing domestic animals. Minxian black fur (MBF) sheep and Small-tail Han (STH) sheep are both Chinese native breeds and are included on the list of protected genetic resources of livestock and poultry in China. MBF sheep are found in the Tao River basin region of Gansu Province in northwestern China. This black fur sheep breed has developed several special traits, including dark skin and hair covering their full body including the visible mucous membranes, lips, and tongues. This pattern is similar to the phenotype of black-boned sheep (Deng et al., 2006) and the dark goat (Ren et al., 2017). The STH sheep is one of the most famous local breeds for its reproductive performance and strong adaptability and is widely distributed in most parts of northern China. We used STH sheep breeds as the control group for their nearly white skin color and white coat color.

Skin color variation is one of the most common examples of phenotypic diversity in animals. Skin color in sheep is predominantly determined by the amount, type, and packaging of melanin polymers. Those polymers are produced by melanocytes and then secreted into keratinocytes (Rees, 2003). Melanocytes in skin are present in hair follicles and in the basal skin layer. However, the numbers of melanocytes vary in these locations (Tobin & Bystryn, 1996). Melanin polymer molecules can have protective or harmful biological functions. Melanin provides protection against DNA-damage from ultraviolet radiation, yet the melanin in pathogenic fungi can aid host infection. Melanin is also a component of a recently described antifungal defense pathway in mammals (Casadevall, 2018). Melanocytes and neighboring cells interact in keratinocytes and fibroblasts to regulate skin color (Choi, Kolbe & Hearing, 2012). The distribution of melanin in the epidermis appears to protect DNA from photodamage, as evidenced by the considerable range of skin colors and corresponding incidence of keratinocyte cancers in humans (Fajuyigbe et al., 2018).

Skin pigmentation in animals is a polygenic trait, and may be influenced by different kinds of multi-gene interactions (Barsh, 1996). The proteins and genes associated with skin color in humans, mice, chickens, and sheep include agouti signaling protein (ASIP) (Lu et al., 1994), melanocyte-stimulating hormone receptor (MSH) (Robbins & Nadeau, 1993), microphthalmia transcription factor (MITF) (Dong, Yoshiaki & Vijayasaradhi, 2002; Vetrini et al., 2004; Wang et al., 2018), tyrosinase (TYR) (Deng et al., 2008; Yu et al., 2017), tyrosinase-related protein-1 (TYRP1) (Chun et al., 2016), dopachrome tautomerase (DCT) (Xue et al., 2018), melanocortin 1 receptor (MC1R) (Fontanesi et al., 2010; Bourgeois et al., 2016; Wolf Horrell, Boulanger & D’Orazio, 2016), solute carrier family 45 member 2 (SLC45A2) (Wang et al., 2016), and others. There are over 100 known color loci in mice and many of them have been cloned and sequenced (Bennett & Lamoreux, 2003).

Many biological mechanisms in sheep skin can be assessed using comparative anatomy, transcriptomics, and genomics (Jablonski, 2004). The transcriptome of the skin of fine and coarse wool sheep breeds in China have been evaluated (Zhang et al., 2017a). Skin transcriptome profiles associated with coat color in sheep (Fan et al., 2013) or goats (Ren et al., 2017) have also been characterized. Melanin in the skin of other species, including koi carp (Luo et al., 2019), raccoon dog (Du et al., 2017), turtle (Si et al., 2019) and mink (Song et al., 2017) has been genetically characterized by sequencing the RNA populations. However, there is a lack of understanding regarding the underlying genetic mechanisms of pigmentation. The dark pigmentation of the MBF sheep represents an opportunity to assess and characterize the genetic and structural properties of skin in this unusual breed when compared to a white breed of sheep. We sought to investigate the genetic mechanisms associated with pigmentation by: (1) physically characterizing skin melanin distribution and other skin characteristics, and (2) assessing the genes expressed in skin using RNA sequencing technology in MBF sheep compared to the white Small-tail Han breed.

## Materials & Methods

### Ethics Statement

This study was approved by the Committee for Animal Ethics of the College of Animal Science and Technology, Gansu Agricultural University (approval number 2019-75). Experiments were conducted in accordance with approved guidelines.

### Animals and tissue sampling

Three healthy MBF males and three STH males one year of age were obtained from specialized sheep farming cooperatives in Min county, Gansu Province, China. Sheep were selected to minimize relatedness. Sheep were fed under the same conditions and were humanely sacrificed. Two adjacent skin samples were obtained from the scapular region of each animal using surgical scissors. One sample was designated for RNA extraction and immediately placed into liquid N and subsequently stored at −80 °C (ULT Freezer Model DW-86L828; Haier Biomedical, Qingdao, China). The other sample was designated for histological examination and fixed in a 4% paraformaldehyde solution.

### Sample histological examination and analysis

Samples were soaked in the 4% paraformaldehyde solution for 3 days, dehydrated in a graded ethanol series, and embedded in paraffin. Samples were partitioned into 6-µm sections using a rotary microtome (Leica RM2265, Germany) and were subjected to haematoxylin and eosin (H&E) and Fontana-Masson (FM) staining. FM staining identified the presence and distribution of melanin (Barbosa et al., 1984; Carriel et al., 2011). H&E staining was conducted to observe morphologic parameters. After staining, slides were scanned using a Panoramic Scanner (P-MIDI; 3DHISTECH Ltd., Hungary). The thickness of the epidermis and the dermis of each sample were measured five times using CaseViewer (C.V 2.0; 3DHISTECH Ltd., Hungary). Fontana-Masson (FM) is a specific staining method used to detect melanin granules, which appear black under the stain. The content of melanin granules was determined by detecting the relative area of the granules in the sample slices. We detected the relative content, rather than the absolute content, using this method. Slide images were analyzed using Image-Pro Plus (6.0; Media Cybernetics, MD, USA) software.

### RNA Extraction, transcriptome library construction, and sequencing

Total RNA in each sample was isolated after pulverizing the sample in liquid N using the RNA simple Total RNA Kit (Tiangen, Beijing, China) according to the manufacturer’s instructions. The concentration and purity of total RNA were detected using a NanoDrop-2000 spectrophotometer (Thermo Scientific, Waltham, MA, USA). All samples exhibited optical density 260/280 ratios from 1.8 to 2.0. RNA integrity was determined by 1.0% agarose gel electrophoresis. Samples were stored at −80 °C for subsequent library construction and sequencing.

Approximately 5 µg RNA per sample was used as input material in constructing the sequencing library. First, ribosomal RNA was removed using an Epicentre Ribo-zero™ rRNA Removal Kit (Epicentre, Madison, WI, USA) and a fragmentation buffer was added to process mRNA into short fragments. The first cDNA strand was synthesized using random hexamer primers and the second-strand cDNA was synthesized using dNTPs, buffer, RNaseH, and DNA polymerase where dTTP were replaced by dUTP. The cDNA fragments were purified using a QIAquick PCR extraction kit (Qiagen, Gmbh, Germany). The fragment ends were repaired and poly-adenosine tails were ligated to sequencing adaptors. Suitable size fragments were selected following agarose gel electrophoresis and were used as templates for polymerase chain reaction (PCR) amplification.

Sequencing was performed using the HiSeq™ 2000 (Illumina, Inc., San Diego, CA, USA) system to create the library.

### Quality control and reads mapping

Raw data were first processed using in-house Perl scripts. Clean data were obtained by removing reads containing adapters, reads containing over 10% of poly-N, and low-quality reads (more than 50% of bases whose Phred scores Q20error rate were less than 5%). The Phred scores and guanine-cytosine (GC) contents of the quality-edited data were calculated and used as metrics in this process. Subsequent analyses were conducted using the quality-edited data.

Reads from RNA sequencing were mapped to the *Ovis aries* genome (Oar_v4.0; https://www.ncbi.nlm.nih.gov/assembly/GCF_000298735.2/). The paired-end clean reads were aligned to the reference genome with HISAT2 (V2.0.4) (Kim, Langmead & Salzberg, 2015) and the assembled transcripts were constructed using String Tie (V1.3.1) (Pertea et al., 2016).

The strands specific parameter was the main parameter setting (–rna-strandness RF) and the name and location of the input and output files were set while all other alignments were set to default parameters. String Tie software was used for assembling and counting the original sequences of reads from each sample. The default settings were used for most parameters, aside from the name and location of the input or output file.

### Screening and identification of differentially expressed genes

String Tie software was used for counting the original sequences of reads from each sample. Then, we calculated the fragments per kilo base of exon model per million reads mapped (FPKM) (Trapnell et al., 2010) of coding genes relative to the expression levels of known genes. We used the R package DESeq (Love, Huber & Anders, 2014) to screen differentially expressed genes. Differentially expressed genes were declared (Audic & Claverie, 1997; Benjamini & Yekutieli, 2001) with fold change thresholds of 2 and FDR controlled at less than 0.05. We created a hierarchically clustered heatmap of known differentially expressed genes using Omicshare software tools (https://www.omicshare.com/tools/Home/Soft/heatmap).

### Enrichment analysis of differentially expressed genes

Known DEGs were used in functional enrichment analysis. We conducted enrichment analysis of differentially expressed genes after annotation (Gene Ontology, 2015) using the online version of omicShare (Gene Denovo Ltd., Guangzhou, China) and the Kyoto Encyclopedia of Genes and Genomes (KEGG) pathways using KOBAS (V3.0) (Mao et al., 2005) software. KOBAS is a widely used gene set enrichment (GSE) analysis tool (KOBAS 3.0, 2019). All genes of the reference genome (the Ovis aries genome -Oar_v4.0) were used as the background gene set while conducting functional enrichment analysis.

### Verification of differentially expressed genes

We verified the reliability and accuracy of the RNA-Seq results using RT-qPCR for eight of the differentially expressed genes. cDNA was synthesized from extracted RNA using a PrimeScript™ RT Reagent kit (Takara, Dalian, China) and stored at −20 °C. The specific primers of these target genes were designed using NCBI Primer-BLAST online software; those sequences and related information are presented in Table 1. The reaction volume for RT-qPCR included 9.5 µl SYBR® Green PCR Master Mix (Takara, Dalian, China), 1 µl cDNA, 1 µl forward and reverse primers, and 7.5 µl RNase free ddH_2_O. Each reaction was performed in triplicate. The relative expression of target genes was calculated using the 2^−ΔΔCt^ method (Livak & Schmittgen, 2001). Glyceraldehyde-3-phosphate dehydrogenase (*GAPDH*) was used as an internal reference gene.

### Statistical analysis

We assessed the differences in melanin content, and the thickness of the epidermis, dermis, and skin of the two breeds were using the two-tailed Student’s *t*-test method with SPSS (IBM, Armonk, NY, USA) software. Results were presented as means and standard errors.

## Results

### Histological characterization

**Figure 1:**
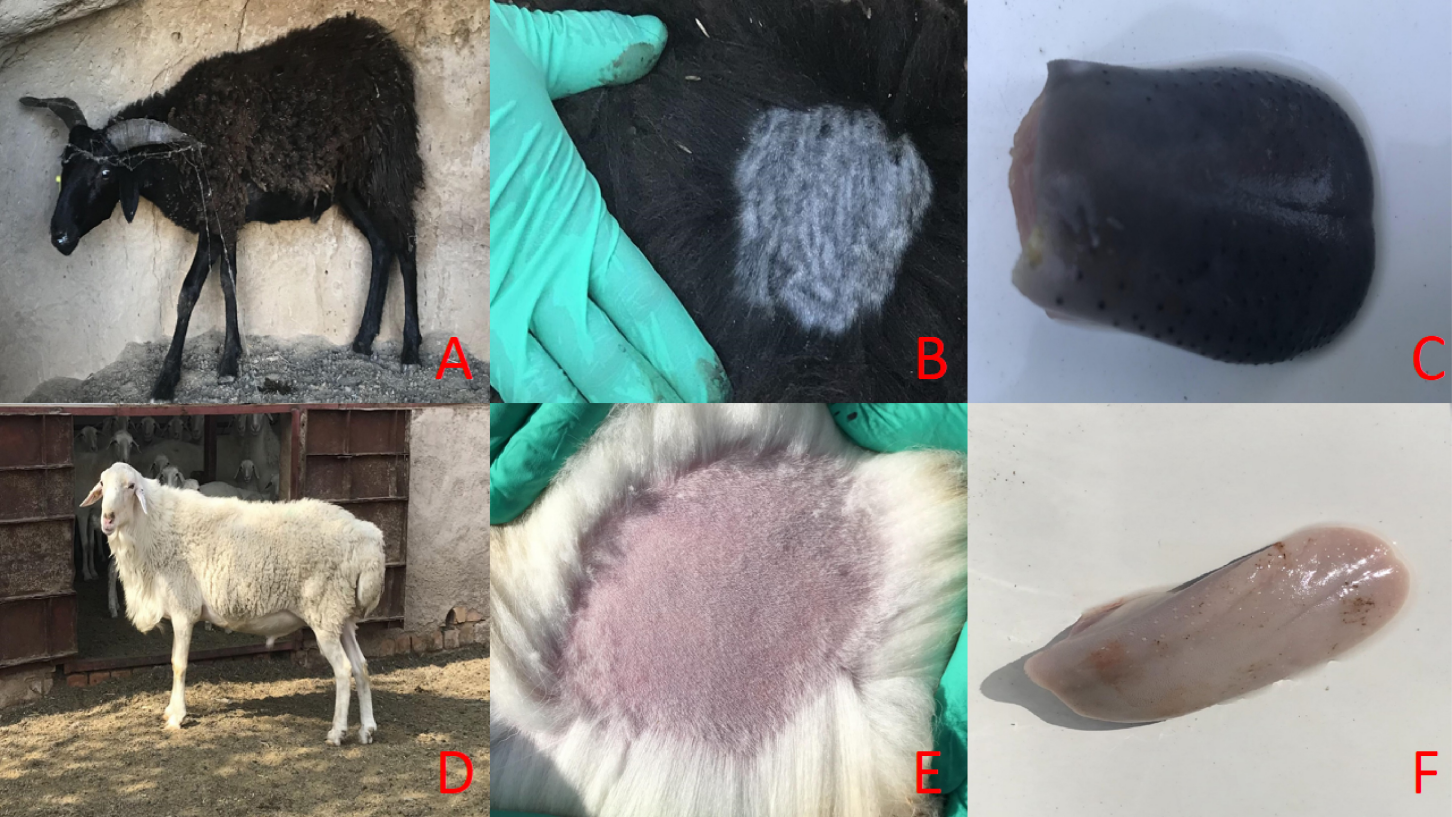
The main phenotypic characteristics of Minxian Black Fur sheep and Small-tail Han sheep. Overall appearance of Minxian Black Fur sheep and Small-tail Han sheep (A and D), Minxian Black Fur sheep have dark skin and tongue(B and C), While Small-tail Han sheep have white skin and tougue (E and F).

**Figure 2:**
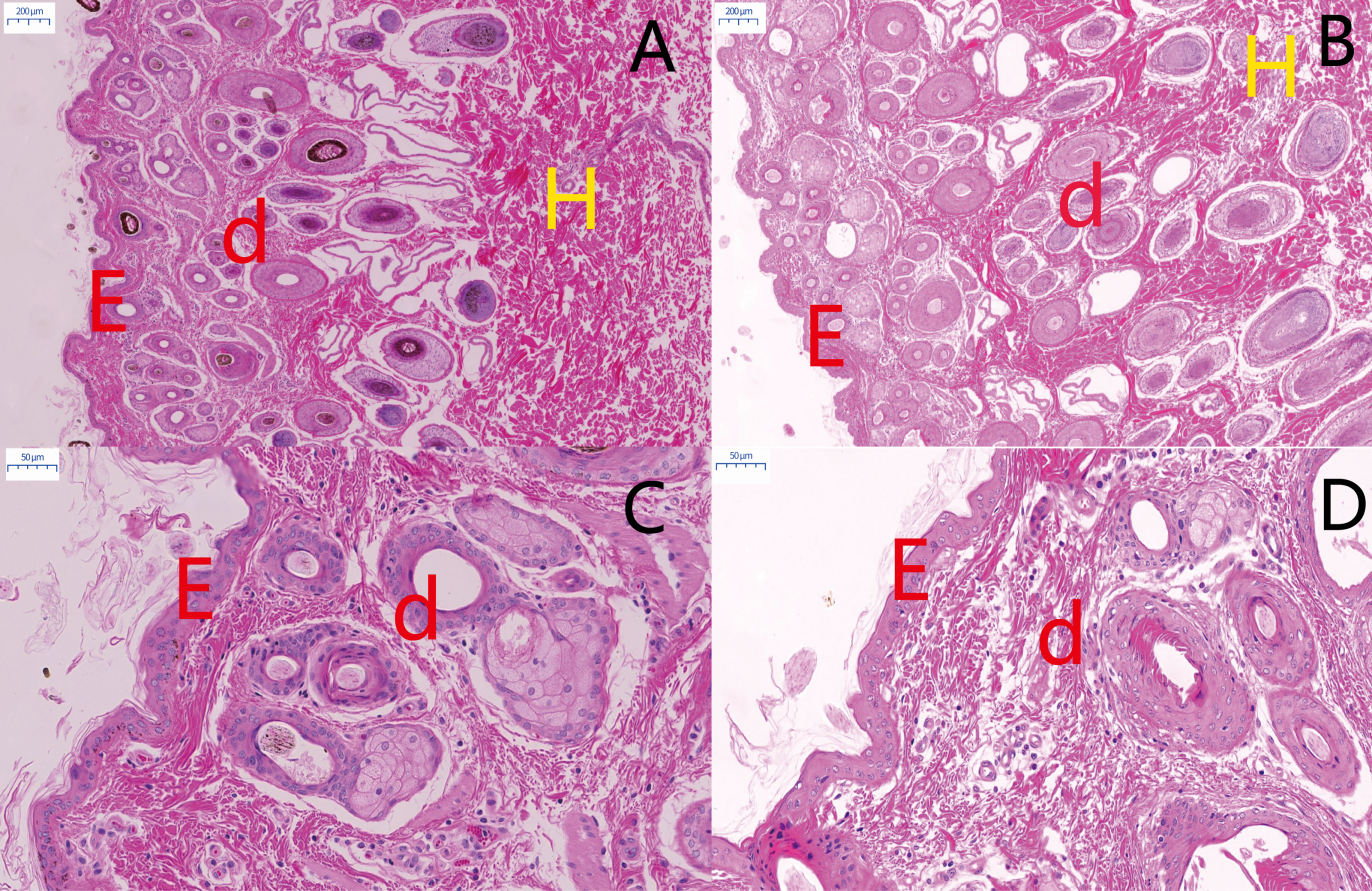
Histological comparison of sheep skin tissue by H&E staining. Morphological comparisons of skin tissues in MBF sheep and STH sheep. The representative images of MBF and STH at a scale of 200 um (A, B); the representative images of MBF and STH at a scale of 50 um (C, D); e, epidermis; d, dermis; h, hypodermis.

**Figure 3:**
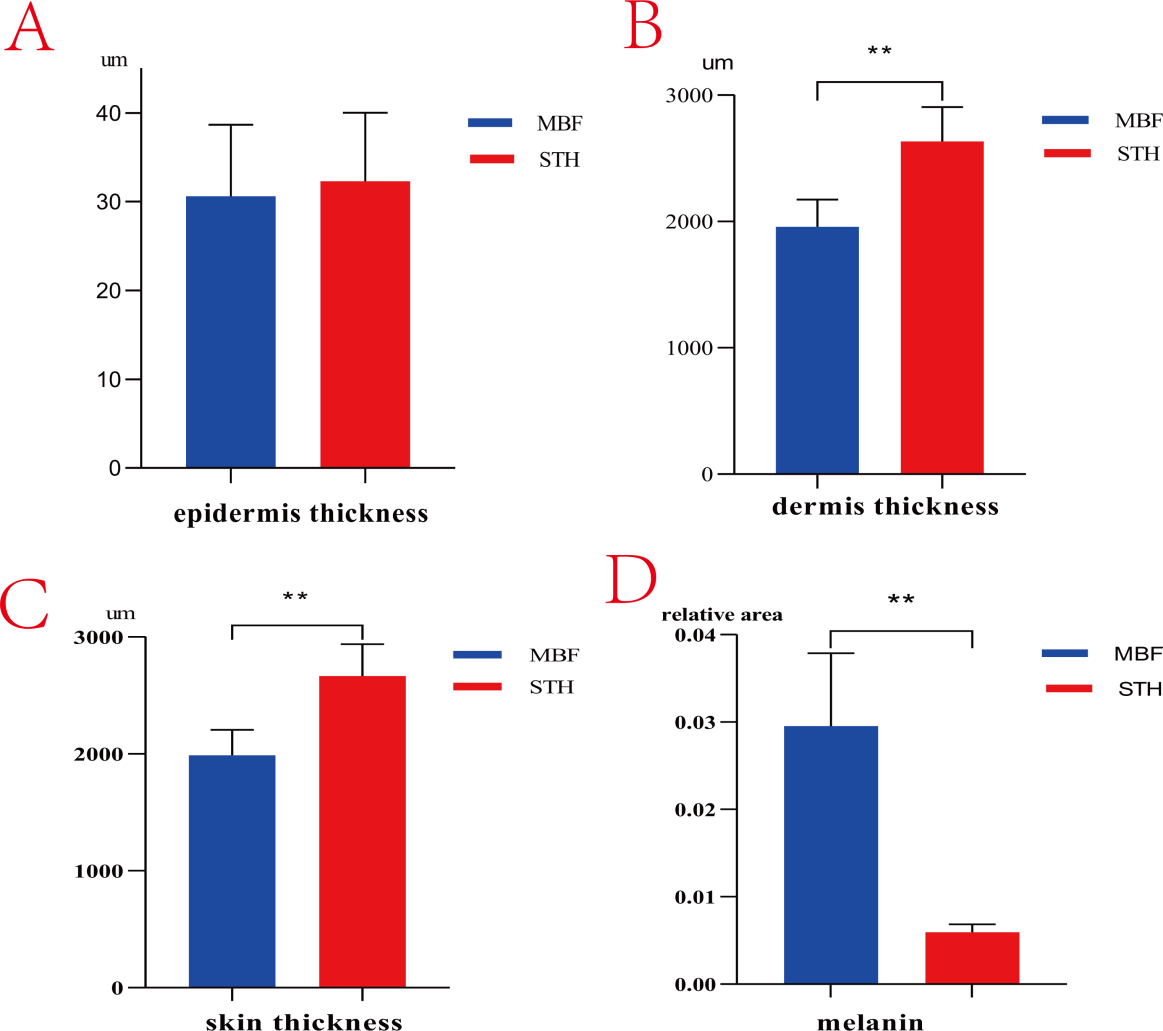
The thickness of skin and melanin content. The results of thickness and melanin content between MBF sheep and STH sheep. The thickness of epidermis (A, *P* > 0.05); The thickness of dermis (B, *P* < 0.01); The thickness of whole skin layer (C, *P* < 0.01); The melanin granules content (D, *P* < 0.01).

There are obvious phenotypic differences in the pigmentation of MBF and STH breeds (Fig. 1). Morphologic features of the skin from the two breeds were similar but differed in the thickness of the dermis (Fig. 2). The thickness of the skin and dermis of MBF samples were lower (*P* < 0.01) than in STH samples, but the thickness of the epidermis did not differ by breed (Figs. 3A, 3B and 3C). The data for the thickness measurements are shown in Table S1.

**Table 1:** Primers used in this study for qRT-PCR.

**Gene**	**Primer sequences (5′–3′)**	**Accession No.**	**Products length (bp)**
*OCA2*	AGGATTTTGGCTCATGCCTCA	XM_004004469.4	140
	GCACCATGACCCTTTTTCTTGTG		
*DCT*	TTCAATCCCCCTGTGGATGC	NM_001130024.1	170
	TCTGGAGTTTCTTCAACTGGCA		
*TYR*	CTTCTTCTCCTCTTGGCAGATCAT	NM_001130027.1	100
	ATTGCGCAGTAATGGTCCCT		
*DDC*	TTCCTTTCTTCGTGGTGGCTA	XM_027968422.1	179
	TGCAAACTCCACGCCATTCA		
*TYRP1*	CGCGGTATTTGATGAATGGCTG	NM_001130023.1	195
	AAACTCCGACCTGGCCATTG		
*MC1R*	GCTGCTGGGTTCCCTTAACT	NM_001282528.1	149
	CCAGCACGTTCTCCACAAGA		
*PMEL*	AGATGCCAACTGCAAAGGGT	XM_012174772.3	187
	AGGGAGCCTGCAGTACCTT		
*TMEM231*	GAACTGCCGAGGGAACAAGA	XM_004014913.4	188
	TTTTAGCCAAAACCCGTGGC		
*GAPDH*	ATGGCCTCCAAGGAGTAAGGT	NM_001190390.1	123
	AGGTCGGGAGATTCTCAGTG		

**Figure 4:**
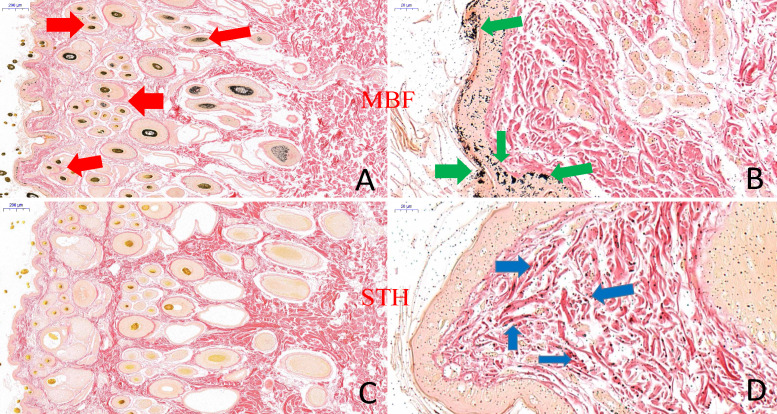
The result of skin tissues Fontana-Masson staining in MBF sheep and STH sheep. Skin tissues Fontana-Masson staining in MBF sheep and STH sheep. The images of MBF sheep (A:200 um; B:20 um); The images of STH sheep (C:200 um; D:20 um). Red, green, and blue arrows indicate the presence of melanin in wool follicles, epidermis, and dermis, respectively.

There were clearly differences between the breeds in the distribution of melanin granules in the skin (Fig. 4). Melanin pigment was distributed across the epidermal layer and wool follicles in MBF samples, but was limited to the dermis in STH samples. There were substantially higher quantities of melanin pigment in the MBF samples (*P* < 0.01) (Fig. 3D) (Table S2). Histological examination and analysis of the skin from two sheep breeds showed that the skin structure of MBF sheep is similar to that of STH sheep, with the exception of the melanin and the thickness of the dermis.

### RNA sequencing and mapping of skin transcriptome

We obtained 103.18 GB clean reads from the six skin libraries after filtering. The total number of reads ranged from 53.84 to 61.81 million. The GC contents of the six samples were within 51.45% to 52.64%, and Q30 scores were greater than 92.62% for each sample. Mapping ratios ranged from 87.40% to 90.57%. Detailed information of RNA sequencing and mapping by sample are listed in Table S3. Samples were further analyzed when the results of sequencing and quality control were good.

### Identification of differentially expressed genes

**Figure 5:**
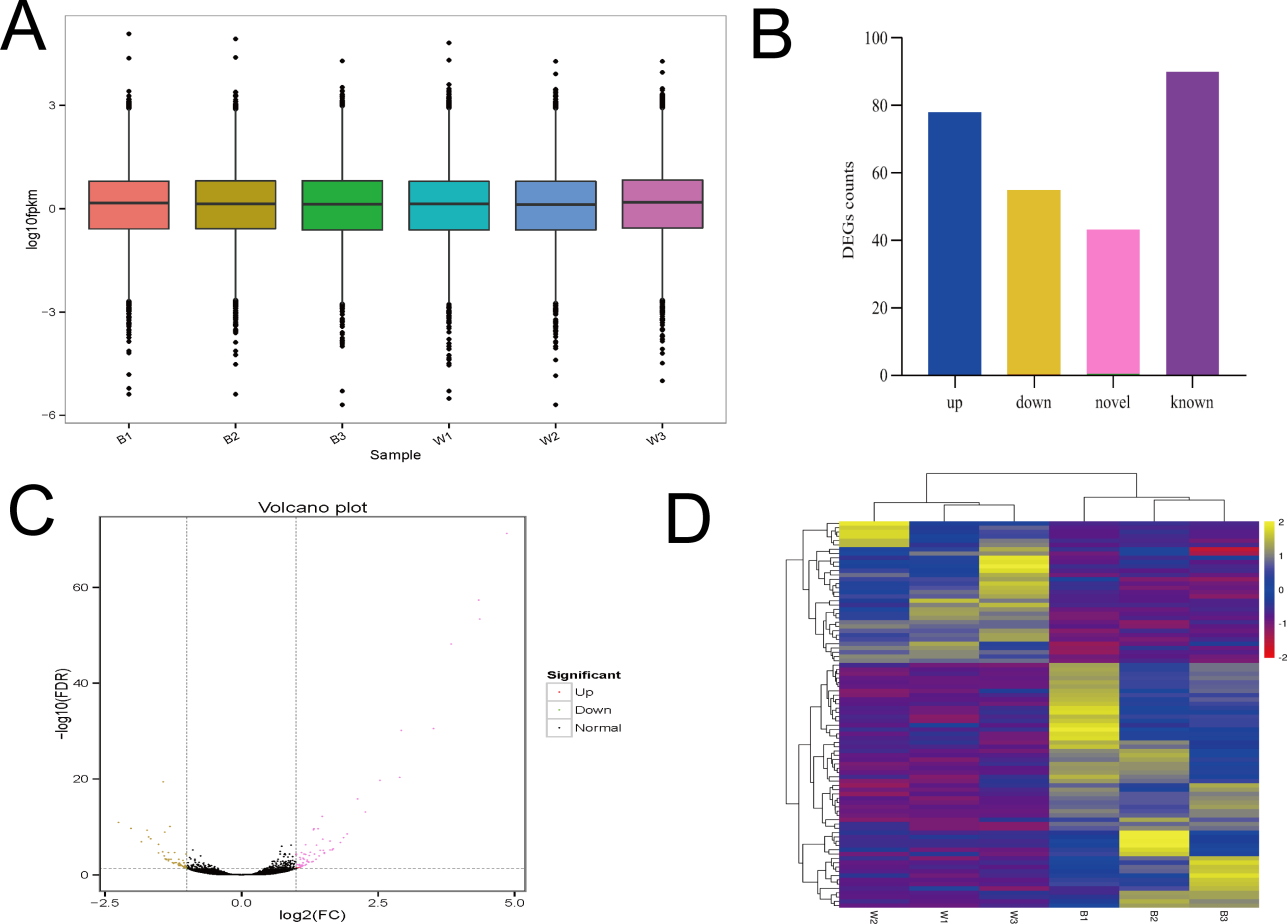
The analysis of Overall gene expression and differentially expressed genes. (A) The overall expression of genes in two breeds of sheep (MBF:B1,B2,B3; STH:W1, W2, W3). (B) Differentially expressed genes between MBF and STH sheep, different colors represent the numbers of upregulated, downregulated, novel, and known DEGs. (C) Volcano plot of differential gene expression in MBF and STH sheep, red and yellow dots indicate upregulated and downregulated genes, respectively. (D) Clustered heatmap of the DEGs in samples of MBF and STH sheep, rows represent DEGs while columns represent different samples.

The six samples had similar overall gene expression levels (Fig. 5A). There were 133 differentially expressed genes identified, with 78 up regulated genes and 55 down regulated genes. Of those differentially expressed genes, there were 90 with known functions, and the remainder were potentially novel genes (Figs. 5B and 5C). Clustered heatmap visualization of the differentially expressed genes revealed two that separated according to the different sheep breeds (Fig. 5D). Additional details of the differentially expressed genes are presented in Table S4. There were less than 150 differentially expressed genes found, and the number of known genes was less than 100, suggesting a much more limited number of candidate genes.

### Functional enrichment analysis of differentially expressed genes

**Figure 6:**
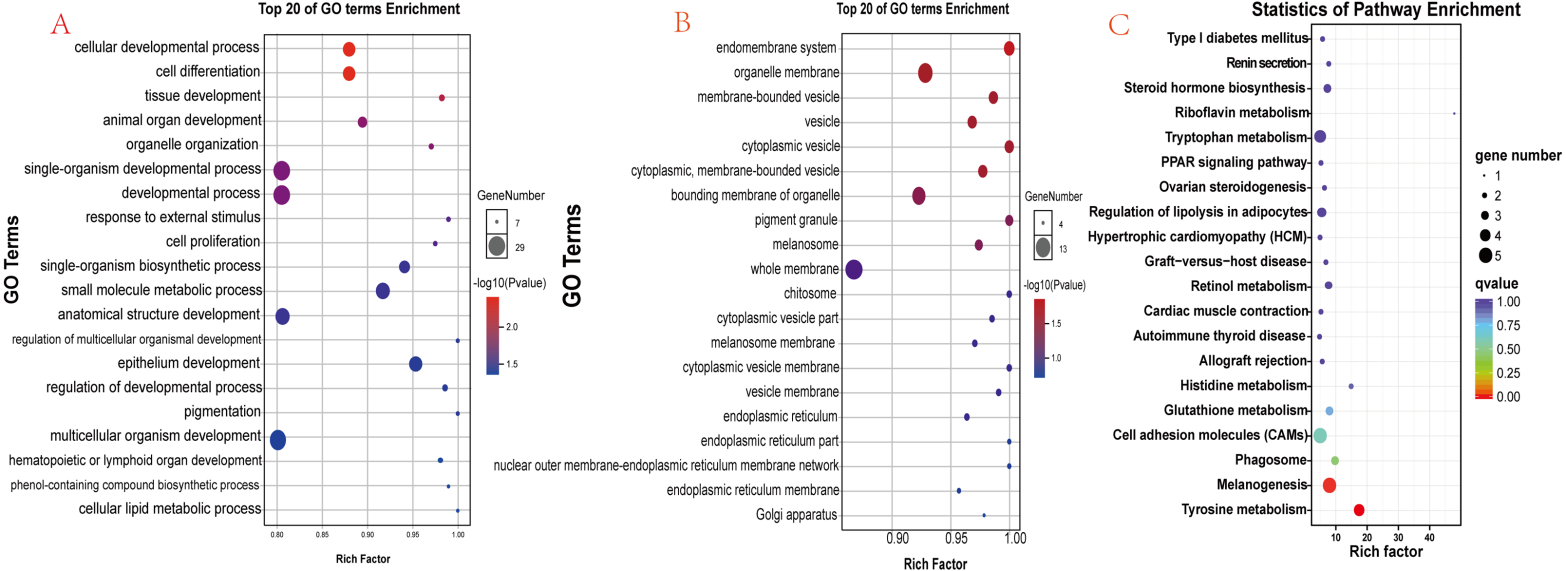
Functional analysis results for differentially expressed genes. (A) The 20 most significant GO terms in the “biological process” category; (B) The 20 most significant GO terms in the “cellular component” category; (C) The 20 most significant pathways identified through KEGG pathway enrichment analysis.

There were 90 known differentially expressed genes that were significantly enriched in 37 GO terms, including 28 in the category “biological process” and 9 in “cellular component” (Table S5). The 20 most significant (lowest *P* values) GO terms in the “biological process” and “cellular component” categories are shown in Figs. 6A and 6B. Some observed significant GO categories were associated with skin and pigmentation. Those included the “melanosome”, “pigment granule”, “tissue development”, and “cell differentiation” categories. The differentially expressed genes were enriched in 114 pathways (Table S6). The 20 most significant pathways are presented in Fig. 6C. Two pathways were involved in the regulation of skin pigmentation (*q* < 0.05): the “tyrosine metabolism” pathway (ko00350, 4 differentially expressed genes) and the “melanogenesis” pathway (ko04916, 5 differentially expressed genes). We found 16 differentially expressed genes in the most significant GO terms and critical signal pathways associated with melanin pigmentation (Table S7). The results demonstrated that enrichment analysis of differentially expressed genes can define their biological function and narrowed the number of candidate genes that may regulate skin.

### RT-qPCR validation of RNA-seq data

**Figure 7:**
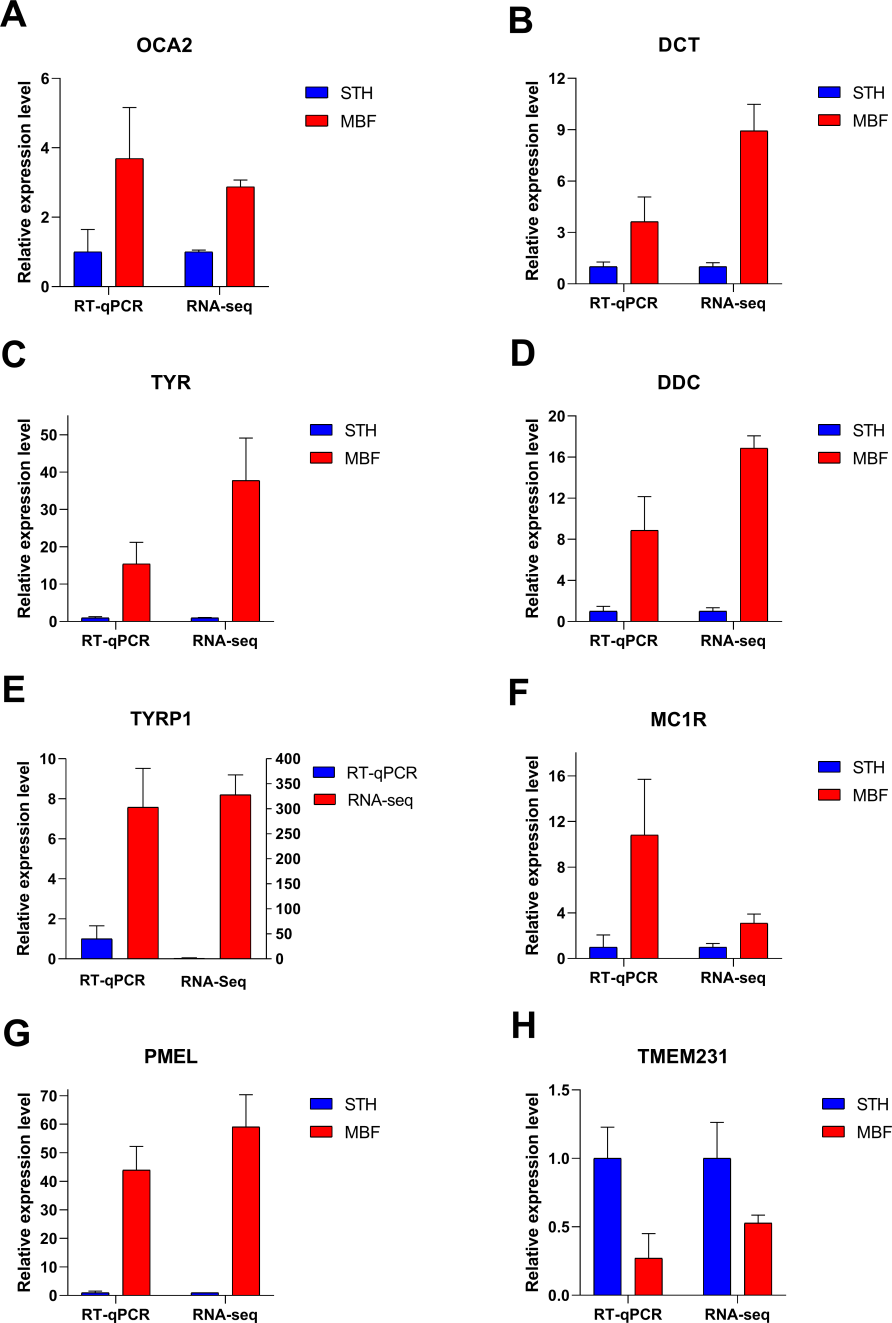
Verification of RT-qPCR for eight differentially expressed genes. FPKM values were used to calculate gene expression in RNA-seq, to normalize the expression of STH group as “1”. Data shown on the *y*-axis represent the fold change. A–H are, respectively, *OCA2, DCT, TYR, DDC, TYRP1, MC1R, PMEL,* and *TMEM231*.

Eight significant differentially expressed genes were identified from the RNA sequencing results for RT-qPCR validation. These differentially expressed genes were chosen from the significantly enriched pathways involved in pigmentation regulation and for their known involvement in the pigmentation of multiple species: *OCA2 melanosomal transmembrane protein* (*OCA2*), *DCT*, *TYR*, *dopa decarboxylase* (*DDC*), *TYRP1*, *MC1R*, *premelanosome protein* (*PMEL*), and *transmembrane protein* (*TMEM231*). The RT-qPCR expression patterns in the two breeds for these genes were consistent with those from RNA sequencing (Fig. 7). Our results demonstrated that the RNA-Seq data and the differentially expressed genes were highly reliable. The identified genes were differentially expressed in the skins of MBF and STH sheep.

## Discussion

Melanin is the most common pigment in most organisms, animals, plants, bacteria, and fungi. The function and mechanism of melanin in the skin and hair have been well-researched. Melanin formation in the skin is induced by long-wave ultraviolet and visible light (Pathak et al., 1962); melanin granules in mammalian melanocytes are formed from the melanosome (Seiji et al., 1963). Melanin granules may move from melanocytes to the cortical cells as a consequence of phagocytosis (Swift, 1964). Melanin pigments in the skin are synthesized and stored in melanosomes, which are functionally and morphologically unique. Melanosomes are distinct organelles similar to lysosomes (Marks & Seabra, 2001). Melanin’s chemical nature is a polyphenolic polymer formed by the oxidative polymerization of phenolic and/or indolic compounds (Bell & Wheeler, 1986). Some studies have shown that melanin may protect against UV and visible light (Singaravelan et al., 2008), antioxidants (Jacobson & Tinnell, 1993; Wolbarsht, Walsh & George, 1981), and radiation (Meredith & Riesz, 2004). Traditional Chinese medicine has promoted chicken soup made with Silkie meat as a curative food as far back as the seventh century. Foods that are naturally rich in melanin including black sesame seed, black fungus, and black bone chicken are considered to have health-giving properties in traditional Chinese medicine. Melanin has been proven to have these functions, but modern beauty standards have promoted lighter skin and current research has focused on inhibiting melanin production (Chen et al., 2014; Li et al., 2017). There are 42 indigenous sheep breeds in China but only a few specialized breeds are used for the production of lambskin and lamb fur (Wei et al., 2015). MBF is a famous sheep breed used for its skin and wool, however, as artificial fur production has improved, the market for natural wool has decreased. The MBF sheep in this study is rich in melanin and can be produced as a specialized breed with health benefits from its meat.

The coloration of the skin and hair in mammals is quite obvious and diverse. Coloration and melanin pigmentation in mammals is an explicit adaptation of natural selection and it is highly related to the average dose of ultraviolet radiation corresponding with the geographic latitude. For example, humans typically have a lighter skin color at latitudes near the polar regions and darker skin in equatorial latitudes (Trivedi & Gandhi, 2017). However, the coloration in MBF sheep may be the result of both natural and artificial selection, for survival in higher altitude environments and stronger ultraviolet rays, as well as a human preference for darker fur. There is a long-held preference for darker wool among the people of the Tao River Basin for its warmth and stain resistance. The evolution of animal coloration can be divided into concealment, communication, and regulation of physiological processes (Caro, 2005). Melanism is a prevailing form of concealment in many organisms (Singaravelan et al., 2010). Communication signaling among mammals includes aposematism, health or reproductive condition, and sexual selection (for example, lionesses prefer to mate with the darkest-maned male in their coalition) (West & Packer, 2002). We believe that there is strong evidence to show that the evolutionary forces responsible for coloration vary greatly between MBF sheep and STH sheep. However, the causes of this variation are not yet understood. Our results may show the gene regulation mechanism implicated in melanin in MBF sheep at the molecular level.

Our study may be the first effort to study melanin pigmentation in this exceptionally pigmented sheep breed. The structural basis of pigment deposition patterns differed markedly in skin samples from these two native Chinese breeds. Skin, epidermal, and dermal thickness in MBF sheep differed from those of STH. Fine wool sheep breeds, such as the Merino, have been shown to have thinner skin thicknesses than other breeds (Zhang et al., 2017a). These results are similar to ours in that MBF sheep have a greater thickness of dermis. Skin color is dependent on the melanin content in the dermis and coat color does not remain static throughout an animal’s life; acute stress or age may cause a depigmentation (Caro, 2005). Technological advances in sequencing have revolutionized evolutionary biology (Sreedharan et al., 2014) and sequencing data have provided a better understanding of biological mechanisms (Alyass, Turcotte & Meyre, 2015). Sequencing of RNA transcripts has been used to investigate mechanisms of pigmentation in different species, and many pigmentation related genes have been identified (Du et al., 2017; Song et al., 2017; Zhang et al., 2017a; Yu et al., 2018; Si et al., 2019). We identified 133 differentially expressed genes, including 90 known genes and 43 new genes. We used software to align the reads to the sheep reference sequence to obtain new genes. The unannotated transcription units which filtering out the sequences that encode too short peptide (only contained a single exon or less than 50 amino acids) were defined as new genes.

Most genes and mutations are identified by their effects on coat color and few are known to alter skin color (Fitch et al., 2003). There are a large number of genes that influence the pigmentation of mammals (Bennett & Lamoreux, 2003) but there are fewer than 15 genes directly associated with skin pigmentation variation in humans (Martin et al., 2017). Some of the identified regulatory genes coincided with our results, including *TYR, TYRP1, OCA2,* and *MC1R*. We found that a small number of critical genes appeared to support the differential regulation of melanin pigmentation in MBF sheep. The candidate genes all come from the significant GO terms and KEGG pathways which regulated and influenced the skin phenotypes. Six differentially expressed genes were components of signal pathways associated with regulating skin pigmentation, including the “tyrosine metabolism” pathway (ko00350) and the “melanogenesis pathway” (ko04916). Those included *TYR, TYRP1, DCT, DDC, MC1R,* and *frizzled class receptor 2* (*FZD2*). These genes were significantly up regulated in MBF and may be the cause of the differences in melanin deposition between the two sheep breeds. Much of our understanding of the molecular biology of melanin formation has been based on *TYR* (Deng et al., 2008; Wang et al., 2014; Yu et al., 2017). Tyrosine peptides are precursors of melanin (Yasunobu, 1957) and tyrosinase catalyzes three reactions in the synthesis of melanin in mammals (Fitzpatrick et al., 1950; Korner & Pawelek, 1982). The high relative expression of genes in the *tyrosinase* family (Guyonneau et al., 2004; Xue et al., 2018), including *DCT* (alias of *TYRP2*)*, TYR*, and *TYRP1* in MBF were similar to that observed in Nanping black-boned sheep (Deng et al., 2008). The melanin and pigmentation in the coat, skin, and muscle in Nanping black-boned sheep appears to be influenced by *TYRP1* (Li et al., 2018), as well as the interaction of *TYR* with *TYRP1* and *DCT* (Rad et al., 2004). Our results indicate that the high expression of the *tyrosinase* gene family, which encode the enzymes that form melanin, may be the major cause of the color phenotype in MBF sheep.

Other differentially expressed genes in our study were associated with pigmentation, including *MC1R, OCA2,* and *PMEL*. *MC1R* is a highly polymorphic gene and a melanocytic Gs protein-coupled receptor. Pigmentation, UV responses, and melanoma risk are regulated by the *MC1R* gene (Wolf Horrell, Boulanger & D’Orazio, 2016). Research has shown that the *MC1R* allelic variations are associated with black or red coat colors in Saudi sheep populations (Mahmoud et al., 2017), which provides the theoretical basis for selecting *MC1R* as the candidate gene in our study. Research has suggested that oculocutaneous albinism type 2 may be caused by mutations in the *OCA2* (pink-eye dilution homolog) gene, resulting in diluted phenotypes (Reissmann & Ludwig, 2013) and sequence variations in *OCA2* showed additional association with eye color (Mengel-From et al., 2010). The differentially expressed *FZD2* gene used in our study is known to be involved in epidermal development (Brennan-Crispi et al., 2018). The expression of *PMEL* is limited to pigmented tissues, including skin melanocytes, uveal melanocytes, and the pigment epithelium of the retina and iris (Charon & Lipka, 2015; Theos et al., 2005). Our analysis identified no more than 20 candidate genes which could potentially regulate skin pigmentation in MBF sheep, including the *tyrosinase* gene family, *MC1R, OCA2,* and *PMEL*. Our results are supported by previous studies although further research should be conducted based on these candidate genes in MBF sheep. Some genes with documented influence on melanin were not differently expressed in these two breeds. Pigment deposition is influenced by *ASIP* (Fontanesi et al., 2009; Fontanesi et al., 2011; Zhang et al., 2017b) and *MITF* (Levy, Khaled & Fisher, 2006; Vachtenheim & Borovansky, 2010; Chen et al., 2018) but they were not differentially expressed in this study. One hundred seventy-one genes have been implicated in pigmentation variability across model organisms (e.g., the Color Genes database: http://www.espcr.org/micemut/), and the key genes that regulate the pigmentation of skin and hair through melanin production are different in different species due to the polygenic effect. Genes that play a regulatory role in other species may not work in sheep. There are no more than 15 genes associated with skin color in humans (Martin et al., 2017) and the number is similar to our result, but the individual genes differ. *MITF* and *ASIP* are not associated with skin color differences in humans but result in coloration differences in MBF sheep. *MITF* is required but is not found in sufficient amounts to induce the expression of melanogenic genes (Gaggioli et al., 2003). The result from Gaggioli et al. (2003) point to the existence of still-unknown regulatory mechanisms that cooperate or synergize with *MITF* to control melanogenic gene expression and melanin synthesis. This also explains why *MITF* is not a differential gene in our study. Research has shown that *MITF* is an activator of tyrosinase family genes and melanin biosynthesis, as it connects melanogenesis with other signaling pathways (Kim, Hwang & Yoon, 2014). We found that *MITF* is not a necessary regulatory factor for melanin synthesis in MBF sheep. *ASIP* is an important endogenous antagonist of melanocortin and *MSH* in vertebrate species (Lu et al., 1994; Jackson et al., 2006), but we did not find that is was associated with skin color.

## Conclusions

The results from skin samples of Minxian black fur sheep showed greater amounts, and a more extensive distribution, of melanin in the skin. The skin samples were thinner than samples taken from white Small-tail Han sheep. A set of candidate genes that regulated melanin biosynthesis, including *TYR, TYRP1, DCT, DDC, MC1R, COA 2* and *FZD2* were identified as differentially expressed in Minxian black fur sheep skin samples. These genes appear to be integral for melanin synthesis through the melanogenesis and tyrosine metabolism signaling processes. Additional skin characterization of the Minxian black fur sheep may present the opportunity to clarify the genetic control of pigmentation.

##  Supplemental Information

10.7717/peerj.11122/supp-1 Table S1 The date of skin thickness in the two breeds of sheep Each sample was tested five times in different positions, B and W refer to the MBF and STH sheep, respectively.

10.7717/peerj.11122/supp-2 Table S2 Date of melanin content The content of melanin granules was determined by detecting the relative area of the granules in the sections of FM staining. The data in the table refers to the number of melanin spots, or the melanin area quantified by the software.

10.7717/peerj.11122/supp-3 Table S3 Statistics and mapping results of RNA-seq data for 6 samples

10.7717/peerj.11122/supp-4 Table S4 Differentially expressed genes identified in Minxian Black Fur sheep and Small-Tail Han sheep by RNA-Seq

10.7717/peerj.11122/supp-5 Table S5 GO enrichment analysis of DEGs in MBF sheep and STH sheep

10.7717/peerj.11122/supp-6 Table S6 KEGG enrichment analysis of DEGs in MBF sheep and STH sheep

10.7717/peerj.11122/supp-7 Table S7 Candidate genes screened from the critical signal pathways and GO terms

## Additional Information and Declarations

### Data Availability

The following information was supplied regarding data availability: Data is available at NCBI SRA: PRJNA650174.
