# Supplementary material for: Comparative transcriptome and histological analyses provide insights into the skin pigmentation in Minxian black fur sheep (Ovis aries)
Source: PeerJ. 2021 Apr 27;9:e11122. doi: 10.7717/peerj.11122 (PMC8086576; doi:10.7717/peerj.11122)
Supplement: Table S1 — Each sample was tested five times in different positions, B and W refer to the MBF and STH sheep, respectively. [file peerj-09-11122-s001.docx]

**Skin thickness** **in the two breeds of sheep**

| unit：um | epodermis thickness | dermis thickness | skin thickness |
| --- | --- | --- | --- |
| B1 | 26.5 | 1501.2 | 1527.7 |
| B1 | 28.3 | 1438.0 | 1466.3 |
| B1 | 30.8 | 1791.2 | 1822.0 |
| B1 | 20.2 | 1800.5 | 1820.7 |
| B1 | 21.6 | 1933.6 | 1955.2 |
| B2 | 29.9 | 1587.3 | 1617.2 |
| B2 | 32.7 | 2061.3 | 2094.0 |
| B2 | 19.3 | 2237.9 | 2257.2 |
| B2 | 42.9 | 2082.9 | 2125.8 |
| B2 | 35.4 | 2306.4 | 2341.8 |
| B3 | 36.4 | 2229.1 | 2265.5 |
| B3 | 28.5 | 2149.2 | 2177.7 |
| B3 | 37.4 | 1887.4 | 1924.8 |
| B3 | 26.9 | 2240.3 | 2267.2 |
| B3 | 41.8 | 2138.6 | 2180.4 |
| W1 | 34.6 | 2379.7 | 2414.3 |
| W1 | 31.9 | 2542.7 | 2574.6 |
| W1 | 26.8 | 2957.7 | 2984.5 |
| W1 | 31.3 | 2866.4 | 2897.7 |
| W1 | 32.7 | 2739.6 | 2772.3 |
| W2 | 41.4 | 2234.7 | 2276.1 |
| W2 | 30.9 | 2825.9 | 2856.8 |
| W2 | 48.9 | 2740.3 | 2789.2 |
| W2 | 20.2 | 2664.2 | 2684.4 |
| W2 | 26.1 | 2752.3 | 2778.4 |
| W3 | 33.5 | 2006.0 | 2039.5 |
| W3 | 31.6 | 2911.8 | 2943.4 |
| W3 | 40.2 | 2741.4 | 2781.6 |
| W3 | 28.2 | 2722.4 | 2750.6 |
| W3 | 26.7 | 2412.0 | 2438.7 |

Each sample was tested five times in different positions, B and W refer to the MBF and STH sheep, respectively.
