# Supplementary material for: Comparative transcriptome and histological analyses provide insights into the skin pigmentation in Minxian black fur sheep (Ovis aries)
Source: PeerJ. 2021 Apr 27;9:e11122. doi: 10.7717/peerj.11122 (PMC8086576; doi:10.7717/peerj.11122)
Supplement: Table S2 — The content of melanin granules was determined by detecting the relative area of the granules in the sections of FM staining. The data in the table refers to the number of melanin spots, or the melanin area quantified by the software. [file peerj-09-11122-s002.docx]

Date of melanin content

|  | | | |
| --- | --- | --- | --- |
| Samples | Melanin area | Section area | Relative ares |
| B1 | 13923 | 431928 | 0.032234539 |
| B2 | 10133 | 279743 | 0.036222533 |
| B3 | 11886 | 588418 | 0.020199926 |
| W1 | 2874 | 583078 | 0.004929015 |
| W2 | 2337 | 353103 | 0.006618465 |
| W3 | 2148 | 339224 | 0.006332099 |

The content of melanin granules was determined by detecting the relative area of the granules in the sections of FM staining. The data in the table refers to the number of melanin spots, or the melanin area quantified by the software.
