# Supplementary material for: Comparative transcriptome and histological analyses provide insights into the skin pigmentation in Minxian black fur sheep (Ovis aries)
Source: PeerJ. 2021 Apr 27;9:e11122. doi: 10.7717/peerj.11122 (PMC8086576; doi:10.7717/peerj.11122)
Supplement: Table S3 [file peerj-09-11122-s003.docx]

Statistics and mapping results of RNA-seq data for 6 samples.

| **Category** | **Paramter** | **MBF sheep** | | | **STH sheep** | | |
| --- | --- | --- | --- | --- | --- | --- | --- |
|  |  | **B1** | **B2** | **B3** | **W1** | **W2** | **W3** |
| reads | clean bases | 17125642736 | 16806772706 | 18294470656 | 17264355444 | 17690230526 | 16001596220 |
|  | clean reads | 57960112 | 57291732 | 61806599 | 58845680 | 60055037 | 53839144 |
|  | GC content (%) | 52.39 | 51.69 | 51.48 | 52.64 | 52.36 | 51.45 |
|  | Q20 (%) | 97.54 | 97.32 | 97.49 | 97.63 | 97.49 | 97.36 |
|  | Q30 (%) | 93.06 | 92.62 | 92.99 | 93.32 | 93.03 | 92.7 |
| mapping to genome | Total reads | 115920224 | 114583464 | 123613198 | 117691360 | 120110074 | 107678288 |
|  | Mapped reads | 103940348 (89.67%) | 101769556 (88.82%) | 111955043 (90.57%) | 102865294 (87.40%) | 108151599 (90.04%) | 97165291 (90.24%) |
|  | Unique mapped | 90326219 (77.92%) | 90899820 (79.33%) | 98850375 (79.97%) | 88984926 (75.61%) | 95610491 (79.60%) | 85520458 (79.42%) |
|  | Multiple mapped | 13614129 (11.74%) | 10869736 (9.49%) | 13104668 (10.60%) | 13880368 (11.79%) | 12541108 (10.44%) | 11644833 (10.81%) |
