# Supplementary material for: Comparative transcriptome and histological analyses provide insights into the skin pigmentation in Minxian black fur sheep (Ovis aries)
Source: PeerJ. 2021 Apr 27;9:e11122. doi: 10.7717/peerj.11122 (PMC8086576; doi:10.7717/peerj.11122)
Supplement: Table S4 [file peerj-09-11122-s004.docx]

Differentially expressed genes identified in Minxian Black Fur sheep and Small-Tail Han sheep by RNA-Seq

| sequence number | gene_name | W1_W2_W3 | B1_B2_B3 | FDR | log2FC | regulated |
| --- | --- | --- | --- | --- | --- | --- |
| 1 | EBF1 | 6.029 | 9.307 | 4.97E-07 | 1.175008135 | up |
| 2 | LGI2 | 0.427 | 0.143 | 0.003557123 | -1.133824755 | down |
| 3 | RBM8A | 9.757 | 1.046 | 1.94E-10 | -2.020389899 | down |
| 4 | NMU | 2.253 | 0.137 | 0.005809476 | -1.156358657 | down |
| 5 | PRKG2 | 0.092 | 0.575 | 0.001796168 | 1.307100771 | up |
| 6 | NOX5 | 2.81 | 7.344 | 0.003361255 | 1.131565534 | up |
| 7 | LOC101112822 | 11.321 | 2.4 | 1.26E-08 | -1.700543441 | down |
| 8 | LOC101103401 | 0.798 | 3.168 | 0.042943541 | 1.01652099 | up |
| 9 | SNAP91 | 0.01 | 0.48 | 0.002304378 | 1.232351103 | up |
| 10 | FABP7 | 3.039 | 20.018 | 1.38E-16 | 2.1303655 | up |
| 11 | ROS1 | 0.02 | 0.174 | 0.035137002 | 1.023985698 | up |
| 12 | FOXO3 | 2.014 | 4.443 | 0.00086257 | 1.203151182 | up |
| 13 | KCNB2 | 0.041 | 0.259 | 0.023018135 | 1.072535014 | up |
| 14 | LOC101113339 | 1105.475 | 445.926 | 0.002794743 | -1.053839348 | down |
| 15 | FABP4 | 566.338 | 212.917 | 8.99E-11 | -1.306979059 | down |
| 16 | ATP12A | 0.716 | 2.991 | 0.015888575 | 1.112852339 | up |
| 17 | DCT | 1.41 | 12.593 | 6.73E-31 | 2.928764487 | up |
| 18 | FZD2 | 0.393 | 1.703 | 0.023018135 | 1.079694648 | up |
| 19 | TSPAN10 | 0.019 | 1.749 | 6.80E-06 | 1.625982108 | up |
| 20 | ADORA1 | 0.923 | 0.459 | 0.003361255 | -1.048416733 | down |
| 21 | SLC2A5 | 0.699 | 0.151 | 0.003557123 | -1.240916164 | down |
| 22 | LOC106990122 | 4.83 | 39.463 | 1.90E-08 | 1.870497567 | up |
| 23 | TMEM231 | 3.237 | 1.711 | 0.000557095 | -1.142462626 | down |
| 24 | MC1R | 0.715 | 2.21 | 0.03378402 | 1.004151115 | up |
| 25 | EPHA3 | 2.37 | 0.528 | 4.89E-10 | -1.726480743 | down |
| 26 | LOC101112590 | 4.178 | 1.308 | 2.31E-05 | -1.332885917 | down |
| 27 | SEPW1 | 69.393 | 25.241 | 3.90E-20 | -1.432768824 | down |
| 28 | ZBTB16 | 2.439 | 5.579 | 2.42E-10 | 1.327930699 | up |
| 29 | TRIM66 | 0.118 | 0.404 | 0.002785364 | 1.127527684 | up |
| 30 | UPK1B | 3.838 | 0.358 | 0.000523064 | -1.391853057 | down |
| 31 | SLC45A2 | 0.123 | 5.25 | 6.53E-49 | 3.843184942 | up |
| 32 | SRRM4 | 0.028 | 0.098 | 0.0116717 | 1.107037935 | up |
| 33 | FAM169B | 0.48 | 1.354 | 0.035062696 | 1.005500852 | up |
| 34 | TRPM1 | 0.013 | 2.78 | 3.63E-54 | 4.36182054 | up |
| 35 | APBA2 | 0.124 | 1.008 | 0.017000564 | 1.113142287 | up |
| 36 | CYP1A1 | 0.201 | 1.712 | 3.95E-06 | 1.677559163 | up |
| 37 | CLMN | 0.425 | 1.203 | 2.45E-05 | 1.102908634 | up |
| 38 | GPX1 | 28.686 | 9.651 | 1.17E-09 | -1.401045968 | down |
| 39 | FKBP5 | 20.092 | 45.035 | 0.000243744 | 1.067729853 | up |
| 40 | LOC101106291 | 5.643 | 1.574 | 0.038708491 | -1.027460864 | down |
| 41 | OVAR-DRB3 | 1.442 | 0.112 | 0.01457999 | -1.076074931 | down |
| 42 | LOC106990117 | 2.39 | 0.869 | 0.017613724 | -1.028141381 | down |
| 43 | ZNF165 | 11.123 | 2.701 | 4.43E-07 | -1.514043765 | down |
| 44 | IRF4 | 0.702 | 2.199 | 0.008850004 | 1.040905048 | up |
| 45 | TYR | 0.361 | 6.199 | 4.71E-21 | 2.897213442 | up |
| 46 | LOC101110550 | 1.213 | 0.048 | 0.002908118 | -1.196383594 | down |
| 47 | METTL12 | 10.55 | 2.901 | 2.94E-08 | -1.659409338 | down |
| 48 | LOC101117809 | 1.126 | 0.165 | 0.012853831 | -1.124129071 | down |
| 49 | PLEKHS1 | 1.098 | 2.444 | 5.90E-05 | 1.179914124 | up |
| 50 | HIVEP3 | 0.073 | 0.352 | 9.44E-05 | 1.243393391 | up |
| 51 | HSBP1L1 | 1.095 | 3.324 | 1.84E-05 | 1.26048347 | up |
| 52 | LAMA1 | 0.16 | 0.854 | 0.027743403 | 1.041904709 | up |
| 53 | SDR42E2 | 0.502 | 1.721 | 0.009468231 | 1.167925676 | up |
| 54 | LOC101116799 | 29.219 | 97.223 | 6.30E-07 | 1.378315107 | up |
| 55 | GPR143 | 0.471 | 3.582 | 8.23E-14 | 2.266672859 | up |
| 56 | SLC38A5 | 0.171 | 0.813 | 0.012890163 | 1.110331671 | up |
| 57 | EDA2R | 0.716 | 0.268 | 0.000965803 | -1.068182525 | down |
| 58 | GALNT15 | 1.37 | 5.908 | 7.62E-06 | 1.500857636 | up |
| 59 | EFHB | 0.656 | 3.366 | 0.031148772 | 1.050732067 | up |
| 60 | LOC101106404 | 7.01 | 3.26 | 4.45E-05 | -1.007213213 | down |
| 61 | PLP1 | 0.972 | 3.065 | 2.42E-10 | 1.403624736 | up |
| 62 | LOC105605903 | 3.235 | 0.671 | 0.011761537 | -1.148383188 | down |
| 63 | C2H9orf84 | 0.176 | 1.527 | 0.000595663 | 1.364022665 | up |
| 64 | CCDC180 | 0.894 | 0.165 | 0.002953385 | -1.255830711 | down |
| 65 | MLANA | 0.005 | 5.677 | 4.86E-72 | 4.854423588 | up |
| 66 | TYRP1 | 0.265 | 86.864 | 0.001697263 | 1.202506307 | up |
| 67 | OCA2 | 1.717 | 5.993 | 3.37E-08 | 1.49387627 | up |
| 68 | MYO7B | 0.443 | 0.092 | 0.000557095 | -1.365392201 | down |
| 69 | DHRS9 | 1.662 | 0.414 | 0.000557095 | -1.322791081 | down |
| 70 | CACNB4 | 0.63 | 1.473 | 4.60E-05 | 1.15026075 | up |
| 71 | KIAA2012 | 1.342 | 0.871 | 2.16E-05 | -1.224144731 | down |
| 72 | PAX3 | 0.004 | 0.201 | 0.002193339 | 1.228857289 | up |
| 73 | CCL20 | 0.883 | 2.772 | 0.000332583 | 1.124013197 | up |
| 74 | PLEK | 0.275 | 0.845 | 0.0116717 | 1.070104352 | up |
| 75 | LOC654331 | 1.728 | 0.325 | 0.031148772 | -1.045864312 | down |
| 76 | SLC4A5 | 0.025 | 0.222 | 0.002215128 | 1.286023434 | up |
| 77 | KERA | 14.401 | 3.876 | 0.003097601 | -1.216018041 | down |
| 78 | PLXNC1 | 1.24 | 3.05 | 4.54E-10 | 1.32171143 | up |
| 79 | LOC101111178 | 82.547 | 20.435 | 1.60E-05 | -1.443636339 | down |
| 80 | LOC101111440 | 2.865 | 0.968 | 0.019696533 | -1.045192037 | down |
| 81 | LOC101104222 | 0.251 | 1.432 | 3.44E-05 | 1.520473322 | up |
| 82 | PMEL | 0.947 | 73.759 | 4.58E-58 | 4.338376355 | up |
| 83 | HAL | 1.079 | 2.296 | 5.02E-05 | 1.001799653 | up |
| 84 | LOC101107948 | 0.75 | 3.813 | 9.47E-08 | 1.619076883 | up |
| 85 | LOC101102973 | 0.887 | 0.36 | 0.000577742 | -1.36767303 | down |
| 86 | DDC | 0.181 | 3.055 | 1.93E-20 | 2.539479757 | up |
| 87 | PDK4 | 11.488 | 36.337 | 0.000557835 | 1.151320544 | up |
| 88 | LOC101102057 | 1.119 | 5.256 | 0.007293237 | 1.188135442 | up |
| 89 | BTNL9 | 0.617 | 1.902 | 7.90E-06 | 1.445189887 | up |
| 90 | LOC101108654 | 0.332 | 51.09 | 0.009162852 | 1.042670276 | up |
| 91 | newGene_1774 | 0 | 0.381004333 | 0.00746709 | 1.063043894 | up |
| 92 | newGene_10299 | 3.449514333 | 0.783882 | 0.002794743 | -1.254494714 | down |
| 93 | newGene_17018 | 0 | 0.212301 | 2.45255E-05 | 1.519014316 | up |
| 94 | newGene_18471 | 2.910580667 | 4.352926667 | 6.37786E-05 | 1.004223755 | up |
| 95 | newGene_20092 | 15.35145833 | 4.039209 | 0.049612611 | -1.005286917 | down |
| 96 | newGene_24002 | 0.605604 | 0.000200667 | 1.33634E-07 | -1.833161281 | down |
| 97 | newGene_26647 | 2.665370667 | 0.949962333 | 0.017005225 | -1.024967157 | down |
| 98 | newGene_28992 | 0.418503667 | 0.889102667 | 5.06862E-05 | 1.337726845 | up |
| 99 | newGene_32018 | 0.423077333 | 1.487361 | 0.0116717 | 1.098571777 | up |
| 100 | newGene_32824 | 0.374626333 | 0.072375 | 0.004091144 | -1.23866363 | down |
| 101 | newGene_35904 | 0.535208333 | 0.159168 | 0.021218101 | -1.057538814 | down |
| 102 | newGene_50772 | 0.422512667 | 0.812055667 | 0.000584138 | 1.081785387 | up |
| 103 | newGene_51721 | 0.645716 | 0.084384667 | 0.001145976 | -1.339655594 | down |
| 104 | newGene_55606 | 47.47530933 | 116.9500373 | 6.33008E-13 | 1.483824046 | up |
| 105 | newGene_58334 | 0.716798333 | 0 | 2.60368E-05 | -1.513470819 | down |
| 106 | newGene_67868 | 1.003267667 | 0.314004 | 0.030505037 | -1.027826646 | down |
| 107 | newGene_67877 | 0.647144333 | 0 | 0.000621116 | -1.283888949 | down |
| 108 | newGene_74592 | 0.456686 | 0 | 0.000125048 | -1.409318082 | down |
| 109 | newGene_94454 | 0.071784667 | 0.429551333 | 0.01980908 | 1.095259296 | up |
| 110 | newGene_95178 | 0.613985333 | 0.164875667 | 0.005726448 | -1.130542189 | down |
| 111 | newGene_96896 | 0 | 0.252177 | 2.96037E-31 | 3.513233677 | up |
| 112 | newGene_104810 | 1.008750333 | 0.372459 | 0.021295433 | -1.008254332 | down |
| 113 | newGene_105158 | 0.655090667 | 0.079613 | 0.000728604 | -1.373399188 | down |
| 114 | newGene_112077 | 9.569150333 | 1.334552333 | 0.034132138 | -1.012655811 | down |
| 115 | newGene_112856 | 0.077611 | 0.702727 | 2.88708E-09 | 1.940252925 | up |
| 116 | newGene_114443 | 0.617448333 | 0.023438667 | 1.11185E-11 | -2.252318011 | down |
| 117 | newGene_114597 | 2.955169 | 1.147077667 | 0.022842173 | -1.016251237 | down |
| 118 | newGene_121944 | 0.614234667 | 1.542757667 | 0.007106087 | 1.013058342 | up |
| 119 | newGene_122749 | 0 | 0.384747333 | 0.009103509 | 1.042504435 | up |
| 120 | newGene_124609 | 0.070214333 | 0.535305667 | 0.018394892 | 1.094166358 | up |
| 121 | newGene_125128 | 0.229573 | 0.725707333 | 0.00976069 | 1.089451027 | up |
| 122 | newGene_129829 | 0.244889667 | 0.679252 | 0.011801969 | 1.067308508 | up |
| 123 | newGene_142891 | 0.21851 | 0.829926333 | 0.01980908 | 1.082606752 | up |
| 124 | newGene_153293 | 0.153987333 | 0.929758667 | 0.000121044 | 1.422488045 | up |
| 125 | newGene_155589 | 0.360264 | 2.762884667 | 6.05391E-06 | 1.660097489 | up |
| 126 | newGene_155671 | 0.551229667 | 1.654103333 | 0.0086344 | 1.108898961 | up |
| 127 | newGene_169888 | 1.131936 | 0 | 0.009670731 | -1.032410957 | down |
| 128 | newGene_174295 | 1.772239333 | 0.759514333 | 0.02716834 | -1.004193513 | down |
| 129 | newGene_174317 | 0.848750333 | 0.241065333 | 0.012853831 | -1.110527141 | down |
| 130 | newGene_180046 | 0.692457667 | 0.033256 | 0.008517629 | -1.107515196 | down |
| 131 | newGene_186655 | 0 | 0.248685333 | 1.72554E-07 | 1.816590444 | up |
| 132 | newGene_198222 | 0.115843333 | 0.455260667 | 0.012520335 | 1.12380466 | up |
| 133 | newGene_207541 | 2.560856333 | 0.906258 | 0.01980908 | -1.033890019 | down |
