# Supplementary material for: Comparative transcriptome and histological analyses provide insights into the skin pigmentation in Minxian black fur sheep (Ovis aries)
Source: PeerJ. 2021 Apr 27;9:e11122. doi: 10.7717/peerj.11122 (PMC8086576; doi:10.7717/peerj.11122)
Supplement: Table S5 [file peerj-09-11122-s005.docx]

**GO enrichment analysis of DEGs in Minxian Black Fur sheep and Small-Tail Han sheep**

| GO ID | GO Term | Ontology | number | per | BgRatio | qvalue | ratio |
| --- | --- | --- | --- | --- | --- | --- | --- |
| GO:0030154 | cell differentiation | BP | 22 | 24.44 | 277 | 1.63E-07 | 0.08 |
| GO:0048869 | Cellular developmental process | BP | 22 | 24.44 | 342 | 1.63E-07 | 0.06 |
| GO:0009888 | tissue development | BP | 11 | 12.22 | 408 | 4.96E-07 | 0.03 |
| GO:0048513 | animal organ development | BP | 17 | 18.89 | 423 | 1.14E-06 | 0.04 |
| GO:0006996 | organelle organization | BP | 10 | 11.11 | 640 | 1.22E-06 | 0.02 |
| GO:0032502 | developmental process | BP | 29 | 32.22 | 640 | 1.22E-06 | 0.05 |
| GO:0044767 | single-organism developmental process | BP | 29 | 32.22 | 375 | 1.44E-06 | 0.08 |
| GO:0008283 | cell proliferation | BP | 9 | 10.00 | 314 | 5.59E-06 | 0.03 |
| GO:0009605 | response to external stimulus | BP | 9 | 10.00 | 3916 | 5.63E-06 | 0.00 |
| GO:0044281 | small molecule metabolic process | BP | 12 | 13.33 | 764 | 6.53E-06 | 0.02 |
| GO:0044711 | single-organism biosynthetic process | BP | 12 | 13.33 | 554 | 1.63E-05 | 0.02 |
| GO:0048856 | anatomical structure development | BP | 25 | 27.78 | 265 | 3.58E-05 | 0.09 |
| GO:0043473 | pigmentation | BP | 8 | 8.89 | 464 | 3.58E-05 | 0.02 |
| GO:0050793 | regulation of developmental process | BP | 8 | 8.89 | 1745 | 5.08E-05 | 0.00 |
| GO:0060429 | epithelium development | BP | 8 | 8.89 | 2871 | 6.74E-05 | 0.00 |
| GO:2000026 | regulation of multicellular organismal development | BP | 8 | 8.89 | 791 | 0.000106645 | 0.01 |
| GO:0007275 | multicellular organism development | BP | 24 | 26.67 | 540 | 0.000153801 | 0.04 |
| GO:0002520 | immune system development | BP | 7 | 7.78 | 494 | 0.00018282 | 0.01 |
| GO:0006082 | organic acid metabolic process | BP | 7 | 7.78 | 1092 | 0.000193418 | 0.01 |
| GO:0018958 | phenol-containing compound metabolic process | BP | 7 | 7.78 | 526 | 0.000231064 | 0.01 |
| GO:0019748 | secondary metabolic process | BP | 7 | 7.78 | 452 | 0.000268136 | 0.02 |
| GO:0019752 | carboxylic acid metabolic process | BP | 7 | 7.78 | 834 | 0.000468769 | 0.01 |
| GO:0030855 | epithelial cell differentiation | BP | 7 | 7.78 | 992 | 0.000588386 | 0.01 |
| GO:0042592 | homeostatic process | BP | 7 | 7.78 | 840 | 0.000588386 | 0.01 |
| GO:0043436 | oxoacid metabolic process | BP | 7 | 7.78 | 306 | 0.000588386 | 0.02 |
| GO:0044255 | cellular lipid metabolic process | BP | 7 | 7.78 | 305 | 0.000648716 | 0.02 |
| GO:0046189 | phenol-containing compound biosynthetic process | BP | 7 | 7.78 | 344 | 0.000648716 | 0.02 |
| GO:0048534 | hematopoietic or lymphoid organ development | BP | 7 | 7.78 | 347 | 0.00106645 | 0.02 |
| GO:0012505 | endomembrane system | CC | 10 | 11.11 | 319 | 0.001236724 | 0.03 |
| GO:0031090 | organelle membrane | CC | 13 | 14.44 | 864 | 0.001236724 | 0.02 |
| GO:0016023 | cytoplasmic, membrane-bounded vesicle | CC | 9 | 10.00 | 864 | 0.001688909 | 0.01 |
| GO:0031410 | cytoplasmic vesicle | CC | 9 | 10.00 | 254 | 0.001692778 | 0.04 |
| GO:0031982 | vesicle | CC | 9 | 10.00 | 304 | 0.001976432 | 0.03 |
| GO:0031988 | membrane-bounded vesicle | CC | 9 | 10.00 | 304 | 0.00198055 | 0.03 |
| GO:0098588 | bounding membrane of organelle | CC | 12 | 13.33 | 279 | 0.00198055 | 0.04 |
| GO:0042470 | melanosome | CC | 8 | 8.89 | 270 | 0.00198055 | 0.03 |
| GO:0048770 | pigment granule | CC | 8 | 8.89 | 623 | 0.002031333 | 0.01 |
