# Supplementary material for: Comparative transcriptome and histological analyses provide insights into the skin pigmentation in Minxian black fur sheep (Ovis aries)
Source: PeerJ. 2021 Apr 27;9:e11122. doi: 10.7717/peerj.11122 (PMC8086576; doi:10.7717/peerj.11122)
Supplement: Table S6 [file peerj-09-11122-s006.docx]

KEGG enrichment analysis of DEGs in Minxian Black Fur sheep and Small-Tail Han sheep

| KEGG n-46(DEGs in the KEGG pathway) KEGG N-8793(All genes in the KEGG pathway) | | | | |
| --- | --- | --- | --- | --- |
| Pathway | ko_ID | DEG-n | Gene |  |
| PI3K-Akt signaling pathway | ko04151 | 2 | 416 | gene12496;gene25290 |
| mRNA surveillance pathway | ko03015 | 1 | 102 | gene1061 |
| GABAergic synapse | ko04727 | 1 | 88 | gene27619 |
| Dilated cardiomyopathy | ko05414 | 1 | 95 | gene4114 |
| Toxoplasmosis | ko05145 | 2 | 134 | gene23081;gene25290 |
| Mucin type O-Glycan biosynthesis | ko00512 | 1 | 35 | gene2789 |
| Cardiac muscle contraction | ko04260 | 1 | 76 | gene4114 |
| RNA transport | ko03013 | 1 | 188 | gene1061 |
| Glutathione metabolism | ko00480 | 2 | 58 | gene22523;gene23063 |
| Cytokine-cytokine receptor interaction | ko04060 | 2 | 252 | gene27665;gene4579 |
| Graft-versus-host disease | ko05332 | 2 | 58 | gene23081;gene23170 |
| Calcium signaling pathway | ko04020 | 1 | 222 | gene27619 |
| Antigen processing and presentation | ko04612 | 2 | 92 | gene23081;gene23170 |
| Oxidative phosphorylation | ko00190 | 1 | 142 | gene13930 |
| Chemokine signaling pathway | ko04062 | 2 | 184 | gene12496;gene4579 |
| Type I diabetes mellitus | ko04940 | 2 | 69 | gene23081;gene23170 |
| Histidine metabolism | ko00340 | 2 | 26 | gene6869;gene7711 |
| Arachidonic acid metabolism | ko00590 | 2 | 91 | gene16830;gene22523 |
| Asthma | ko05310 | 1 | 45 | gene23081 |
| Carbohydrate digestion and absorption | ko04973 | 1 | 43 | gene16012 |
| Rap1 signaling pathway | ko04015 | 1 | 257 | gene16830 |
| Drug metabolism - cytochrome P450 | ko00982 | 1 | 71 | gene23063 |
| Systemic lupus erythematosus | ko05322 | 1 | 155 | gene23081 |
| Melanogenesis | ko04916 | 5 | 129 | gene14017;gene15197;gene17642;gene23805;gene3540 |
| Neurotrophin signaling pathway | ko04722 | 1 | 127 | gene12496 |
| Non-small cell lung cancer | ko05223 | 1 | 58 | gene12496 |
| Alcoholism | ko05034 | 1 | 176 | gene7711 |
| Phagosome | ko04145 | 3 | 205 | gene23081;gene23170;gene9185 |
| Melanoma | ko05218 | 1 | 108 | gene12496 |
| Longevity regulating pathway | ko04211 | 1 | 101 | gene12496 |
| Rheumatoid arthritis | ko05323 | 2 | 112 | gene23081;gene4579 |
| FoxO signaling pathway | ko04068 | 1 | 140 | gene12496 |
| Signaling pathways regulating pluripotency of stem cells | ko04550 | 1 | 168 | gene15197 |
| Long-term depression | ko04730 | 1 | 69 | gene10897 |
| Influenza A | ko05164 | 1 | 192 | gene23081 |
| EGFR tyrosine kinase inhibitor resistance | ko01521 | 1 | 92 | gene12496 |
| Renin secretion | ko04924 | 2 | 77 | gene10897;gene15617 |
| AMPK signaling pathway | ko04152 | 1 | 125 | gene12496 |
| cAMP signaling pathway | ko04024 | 1 | 228 | gene15617 |
| Proteoglycans in cancer | ko05205 | 1 | 251 | gene15197 |
| Sphingolipid signaling pathway | ko04071 | 1 | 124 | gene15617 |
| Measles | ko05162 | 1 | 152 | gene9185 |
| Platinum drug resistance | ko01524 | 1 | 86 | gene23063 |
| Thyroid hormone synthesis | ko04918 | 1 | 81 | gene22523 |
| Biosynthesis of unsaturated fatty acids | ko01040 | 1 | 27 | gene18356 |
| ECM-receptor interaction | ko04512 | 1 | 94 | gene25290 |
| Hippo signaling pathway | ko04390 | 1 | 176 | gene15197 |
| Small cell lung cancer | ko05222 | 1 | 93 | gene25290 |
| Endocytosis | ko04144 | 1 | 285 | gene23170 |
| Endometrial cancer | ko05213 | 1 | 54 | gene12496 |
| Metabolism of xenobiotics by cytochrome P450 | ko00980 | 2 | 78 | gene21632;gene23063 |
| Acute myeloid leukemia | ko05221 | 1 | 57 | gene19075 |
| Autoimmune thyroid disease | ko05320 | 2 | 84 | gene23081;gene23170 |
| Neuroactive ligand-receptor interaction | ko04080 | 2 | 307 | gene15617;gene17642 |
| Regulation of actin cytoskeleton | ko04810 | 1 | 264 | gene23170 |
| Phenylalanine metabolism | ko00360 | 1 | 27 | gene7711 |
| Epstein-Barr virus infection | ko05169 | 1 | 214 | gene23170 |
| Ovarian steroidogenesis | ko04913 | 2 | 62 | gene16830;gene21632 |
| Intestinal immune network for IgA production | ko04672 | 1 | 65 | gene23081 |
| Cell adhesion molecules (CAMs) | ko04514 | 4 | 217 | gene21632;gene15617;gene23081;gene23170 |
| Adrenergic signaling in cardiomyocytes | ko04261 | 2 | 158 | gene23170;gene4114 |
| Allograft rejection | ko05330 | 2 | 70 | gene23081;gene23170 |
| Fatty acid metabolism | ko01212 | 1 | 52 | gene18356 |
| Morphine addiction | ko05032 | 1 | 94 | gene15617 |
| Spliceosome | ko03040 | 1 | 162 | gene1061 |
| Cocaine addiction | ko05030 | 1 | 55 | gene7711 |
| Viral carcinogenesis | ko05203 | 1 | 233 | gene23170 |
| Estrogen signaling pathway | ko04915 | 1 | 108 | gene22850 |
| cGMP-PKG signaling pathway | ko04022 | 2 | 185 | gene10897;gene15617 |
| Basal cell carcinoma | ko05217 | 1 | 80 | gene15197 |
| HTLV-I infection | ko05166 | 3 | 319 | gene15197;gene23081;gene23170 |
| Dopaminergic synapse | ko04728 | 1 | 136 | gene7711 |
| PPAR signaling pathway | ko03320 | 2 | 76 | gene12393;gene13390 |
| Fatty acid elongation | ko00062 | 1 | 28 | gene18356 |
| Aldosterone synthesis and secretion | ko04925 | 1 | 94 | gene22850 |
| mTOR signaling pathway | ko04150 | 1 | 184 | gene15197 |
| Steroid hormone biosynthesis | ko00140 | 3 | 82 | gene16830;gene18356;gene21632 |
| Amphetamine addiction | ko05031 | 1 | 70 | gene7711 |
| Retinol metabolism | ko00830 | 3 | 75 | gene21632;gene4011;gene6720 |
| Arrhythmogenic right ventricular cardiomyopathy (ARVC) | ko05412 | 1 | 71 | gene4114 |
| Ribosome | ko03010 | 2 | 279 | gene7711;gene12010 |
| Olfactory transduction | ko04740 | 2 | 1084 | gene10897;gene24002 |
| MicroRNAs in cancer | ko05206 | 1 | 189 | gene16830 |
| Oxytocin signaling pathway | ko04921 | 1 | 170 | gene4114 |
| Longevity regulating pathway - multiple species | ko04213 | 1 | 72 | gene12496 |
| Chemical carcinogenesis | ko05204 | 2 | 88 | gene21632;gene23063 |
| Amoebiasis | ko05146 | 1 | 118 | gene25290 |
| Pathways in cancer | ko05200 | 3 | 472 | gene15197;gene19075;gene25290 |
| Salivary secretion | ko04970 | 1 | 106 | gene10897 |
| Circadian entrainment | ko04713 | 1 | 117 | gene10897 |
| Serotonergic synapse | ko04726 | 1 | 120 | gene7711 |
| Prolactin signaling pathway | ko04917 | 1 | 89 | gene12496 |
| MAPK signaling pathway | ko04010 | 2 | 302 | gene23063;gene4114 |
| Wnt signaling pathway | ko04310 | 1 | 168 | gene15197 |
| Hematopoietic cell lineage | ko04640 | 1 | 103 | gene7711 |
| TNF signaling pathway | ko04668 | 1 | 112 | gene4579 |
| Staphylococcus aureus infection | ko05150 | 1 | 80 | gene23081 |
| Regulation of lipolysis in adipocytes | ko04923 | 3 | 61 | gene10897;gene13390;gene15617 |
| Axon guidance | ko04360 | 2 | 182 | gene1819;gene6276 |
| Transcriptional misregulation in cancer | ko05202 | 1 | 188 | gene19075 |
| Tyrosine metabolism | ko00350 | 4 | 45 | gene14017;gene23805;gene3540;gene7711 |
| Ras signaling pathway | ko04014 | 1 | 280 | gene19075 |
| Tryptophan metabolism | ko00380 | 2 | 51 | gene21632;gene7711 |
| Viral myocarditis | ko05416 | 2 | 89 | gene23081;gene23170 |
| Focal adhesion | ko04510 | 1 | 223 | gene25290 |
| Tuberculosis | ko05152 | 2 | 211 | gene23081;gene9185 |
| Gap junction | ko04540 | 1 | 111 | gene10897 |
| Hypertrophic cardiomyopathy (HCM) | ko05410 | 1 | 81 | gene4114 |
| Riboflavin metabolism | ko00740 | 1 | 4 | gene23805 |
| Bile secretion | ko04976 | 1 | 101 | gene5995 |
| Leishmaniasis | ko05140 | 1 | 91 | gene23081 |
| Inflammatory bowel disease (IBD) | ko05321 | 1 | 74 | gene23081 |
| Platelet activation | ko04611 | 1 | 134 | gene10897 |
| Herpes simplex infection | ko05168 | 2 | 231 | gene23081;gene23170 |
