# Supplementary material for: Comparative transcriptome and histological analyses provide insights into the skin pigmentation in Minxian black fur sheep (Ovis aries)
Source: PeerJ. 2021 Apr 27;9:e11122. doi: 10.7717/peerj.11122 (PMC8086576; doi:10.7717/peerj.11122)
Supplement: Table S7 [file peerj-09-11122-s007.docx]

Candidate genes screened from the critical signal pathways and GO terms

| GO terms or KEGG pathways | Gene name |
| --- | --- |
| GO:0043473 (pigmentation) | DCT |
|  | SLC45A2 |
|  | TYR |
|  | GPR143 |
|  | TYRP1 |
|  | OCA2 |
|  | PAX3 |
|  | PMEL |
| GO:0060429 (epithelium development) | FZD2 |
|  | TMEM231 |
|  | UPK1B |
|  | GPX1 |
|  | LAMA1 |
|  | TYRP1 |
|  | OCA2 |
|  | PAX3 |
| GO:0042470 (melanosome) | DCT |
|  | SLC45A2 |
|  | TYR |
|  | GPR143 |
|  | MLANA |
|  | TYRP1 |
|  | OCA2 |
|  | PMEL |
| GO:0048770 (pigment granule) | DCT |
|  | SLC45A2 |
|  | TYR |
|  | GPR143 |
|  | MLANA |
|  | TYRP1 |
|  | OCA2 |
|  | PMEL |
| ko00350 (Tyrosine metabolism) | DCT |
|  | DDC |
|  | TYRP1 |
|  | TYR |
| ko04916 (Melanogenesis) | DCT |
|  | FZD2 |
|  | MC1R |
|  | TYR |
|  | TYRP1 |

Some genes were duplicated in GO terms and KEGG pathways. We screened a total of 15 candidate genes regulating skin pigmentation in Minxian Black Fur sheep.
